# Ionomic Signatures of Olive Trees Affected by Quick Decline Syndrome

**DOI:** 10.3390/plants14182834

**Published:** 2025-09-11

**Authors:** Giorgio Mariano Balestra, Mauro Giordani, Eleonora Coppa, Daniele Schiavi, Stefania Astolfi

**Affiliations:** Department of Agriculture and Forest Sciences (DAFNE), University of Tuscia, 01100 Viterbo, Italy; mauro.giordani@unitus.it (M.G.); ecoppa@unitus.it (E.C.); schiavi@unitus.it (D.S.)

**Keywords:** Olive Quick Decline Syndrome (OQDS), *Xylella fastidiosa* subsp. *pauca*, leaf nutrient profile, ionome, sulfur metabolism, thiol compounds, plant defense mechanisms

## Abstract

Olive Quick Decline Syndrome (OQDS), caused by the bacterium *Xylella fastidiosa*, subsp. *pauca*, has devastated olive groves in Italy’s Apulia region since 2013. Despite significant scientific progress, the solution remains elusive. This study investigated the link between olive tree nutritional status and OQDS severity, aiming to uncover potential mitigation strategies. We analyzed leaf nutrient profiles from olive trees in naturally infected areas, categorizing them as asymptomatic (AS), mildly symptomatic (MS), or severely symptomatic (SS). Distinct nutritional differences were observed across these groups. The integration of univariate statistical analysis, hierarchical clustering, and Principal Component Analysis (PCA) revealed a complex relationship between plant nutritional status and disease progression. Notably, the PCA results highlighted the importance of sulfur metabolism, suggesting its role in olive trees’ defense mechanisms and metabolic responses to OQDS. These results provide promising evidence with potential application for dealing with OQDS, and the question of whether plant nutritional status plays a role in the development of OQDS symptoms deserves to be further examined in depth.

## 1. Introduction

The olive groves of southern Apulia (Italy), particularly the Salento peninsula, encompassing the provinces of Lecce, Brindisi, and Taranto, face a significant threat from Olive Quick Decline Syndrome (OQDS). This devastating disease manifests as leaf scorch and dieback of twigs and branches. First observed in 2008 along a limited stretch of the Ionian coast in Lecce [1], the causal agent of OQDS was officially identified in 2013 [1,2] as the quarantine bacterium *Xylella fastidiosa* subsp. *pauca* strain ST53 [3,4]. The infection starts in the leaves and then spreads systemically to the twigs, branches, trunk, and roots, usually causing the death of the entire olive tree within two to four years [1,5,6,7,8]. This disease is a major concern for Italian olive cultivation, particularly in the Apulia region, which accounts for approximately half of the nation’s olive oil production. Unfortunately, the containment measures implemented to date have not achieved the desired results, as the designated “demarcated areas” have progressively shifted northward by about 70 km since 2015 [9,10]. The progression of the disease is inconsistent, with some areas being affected more severely than others. Significant variability in symptom intensity is observed even between adjacent groves or within small areas. A comprehensive investigation to determine whether the presence of *Xylella fastidiosa* correlates with observed symptoms across large areas has not yet been conducted due to numerous concurrent factors that influence disease expression and progression, including the biology of the vectors, such as *Philaenus spumarius* and *Philaenus italosignus*, members of the family Aphrophoridae within the suborder Auchenorrhyncha, and *Neophilaenus campestris* (order Hemiptera, superfamily Cercopoidea, family Aphrophoridae); the genomic traits of susceptible and tolerant hosts; human interventions (e.g., phytosanitary measures and agronomical practices); and climate [11,12,13]. Since 2013, over 500 studies (www.scopus.com) have explored various aspects of *X. fastidiosa*, including its genomic structure, origin, detection, host range, and the role of insect vectors [14]. While some research has investigated innovative approaches to control the pathogen, such as nanotechnology, peptides, and phages [15,16,17,18], relatively few studies have focused on the agroecosystem conditions of olive groves or the relationship between the plants’ nutritional status and the development of OQDS symptoms [19]. Managing the mineral nutrient status of plants can be a crucial strategy for mitigating the effects of various abiotic and biotic stresses [20]. Subtle environmental changes, such as those in temperature or nutrient availability, can alter the balance of plant–pathogen interactions, favoring either the host or the pathogen [21]. Specifically, the plant’s nutritional status can influence its susceptibility or resistance to disease. In this context, the ionome, the complete profile of all mineral nutrients and trace elements in an organism [22], offers a valuable tool for understanding the conditions that promote stress resistance, tolerance, or a reduction in specific disease symptoms. Research has demonstrated the significant role of mineral elements for *X. fastidiosa* both in vitro and during in planta infection [23]. Ionomic studies on various crops, including grapevine, citrus, and *Nicotiana tabacum*, have consistently shown significant differences in mineral nutrient concentrations between healthy and infected plants, as well as between symptomatic and asymptomatic leaves [24,25]. This aligns with fundamental principles of plant pathology, which recognize that plant–pathogen interactions are profoundly influenced by abiotic factors [26] such as the nutritional status of the plant [27] and the surrounding environment [28]. For agricultural crops, this environment encompasses the macroclimate, microclimate, and crop-specific climate, as well as the soil and the conditioning effects of cultivation practices and the plants’ associated microbiome [29]. A recent study on olive trees conducted in Salento proposed that high manganese (Mn) levels might help protect olive trees against *Xylella fastidiosa* by counteracting deficits caused by bacterial infection [7]. Conversely, another study in the same region found low copper (Cu) content in the leaves of infected olive trees, suggesting that this deficiency may be an unusual physiological response linked to the infection itself [30]. Crucially, the role of sulfur (S) nutrition in plant defense mechanisms is increasingly recognized as pivotal [31,32]. S-containing compounds are actively involved in a wide array of defense responses against both biotic and abiotic stresses [33,34]. Therefore, understanding the dynamics of S assimilation in the context of OQDS could be of paramount importance.

This research, conducted in the Apulia region of Italy, investigated the potential correlation between varying degrees of OQDS symptoms and the concentration of mineral nutrients in olive plants. Given the critical role of S in plant defense, a particular emphasis was placed on its involvement in the OQDS pathosystem. We hypothesized that a close relationship might exist between OQDS symptoms and the rate of S assimilation. Consequently, we assessed changes in thiol levels, key sulfur-containing defense molecules, as well as the activity of O-acetylserine(thiol)lyase (OASTL), the final and regulatory enzyme in the S assimilation pathway. The findings of this investigation into S metabolism promise to provide critical insights into the olive tree’s defense responses to *Xylella fastidiosa* infection and could inform novel disease management strategies.

## 2. Materials and Methods

### 2.1. Experimental Farm, Agronomic Management, and Leaf Sampling

**Figure 1:**
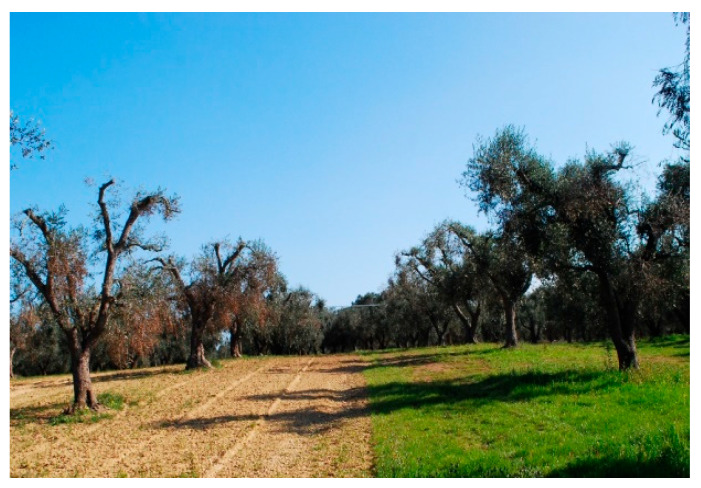
Torchiarolo (BR): olives with severe symptoms and olives with mild symptoms in two contiguous fields (Loc. Case Bianche); March 2017 (40.506313, 18.068236).

This study was conducted in the Apulia region, Italy, in olive groves with ungrafted, approximately 50-year-old Cellina di Nardò trees. We selected orchards with a varying incidence of OQDS symptoms to investigate the relationship between the plants’ condition, the cultivation environment, and disease expression [35,36]. Five plants, displaying different OQDS symptom severity, were identified from three private farms situated within the “Infected area” designated by Italian National Phytosanitary Service: San Ligorio (municipality of Lecce), Torchiarolo (province of Brindisi), and Alezio (province of Lecce). The selection of two geographically distant sites was a strategic decision to avoid localized biases and to gain a more representative understanding of the disease. By including olive groves from municipalities with different histories of pathogen exposure, Gallipoli representing an earlier epidemic front and Torchiarolo a more recent one, we were able to analyze the host’s molecular response across different stages of the disease cycle and identify mechanisms consistent across varied environmental backgrounds. The sampled plants were indeed certified as positive for *Xylella fastidiosa* by the Apulian Regional Phytosanitary Service in 2015, 2015, and 2014, respectively [36,37,38]. The entire investigated region exhibits the occurrence of canopy desiccation; however, the pattern of symptom expression remains irregular, with severely affected areas often adjacent to olive groves displaying milder symptoms or even no visible desiccation (Figure 1). In September 2017, we evaluated the symptoms of OQDS by assessing the percentage of wilted and affected branches within the canopy. Trees were then classified using a five-class severity scale:

1. No wilted branches;
2. Slightly wilted branches (up to 25% of the canopy);
3. Moderately wilted branches (26–50% of the canopy);
4. Severely wilted branches (51–75% of the canopy);
5. Highly severe wilted branches (over 76% of the canopy).

**Figure 2:**
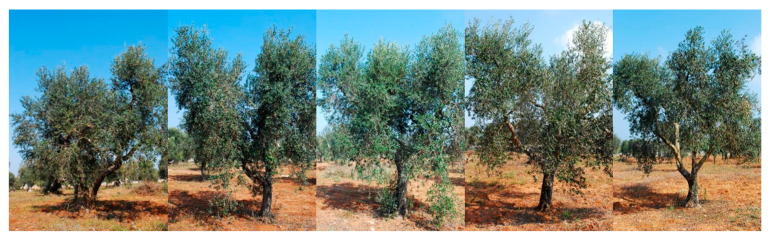
Olive trees infected by *Xylella fastidiosa* in San Ligorio, showing no symptoms (AS) in 2018 (GPS coordinates 40.388690, 18.220066).

**Figure 3:**
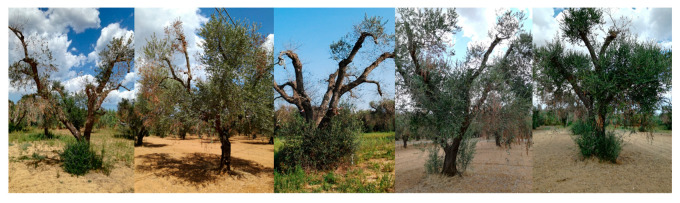
Olive trees infected by *Xylella fastidiosa* in Torchiarolo, showing milder symptoms (MS) in 2018 (GPS coordinates 40.506313, 18.068236).

**Figure 4:**
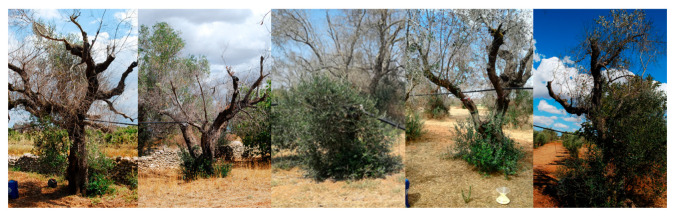
Olive trees infected by *Xylella fastidiosa* in Alezio, showing severe symptoms (SS) in 2018 (GPS coordinates 40.070450, 18.043294).

Across the three farms, the characteristic symptoms of OQDS—leaf scorch, twig, and branch dieback—manifested with varying degrees of severity. Specifically, the olive grove located in the municipality of San Ligorio (GPS coordinates 40.388690, 18.220066), designated as the AS site, exhibited no visible branch desiccation attributable to OQDS (Figure 2); the field in Torchiarolo (GPS coordinates 40.506313, 18.068236), designated as the MS site, displayed mild symptomatology, with approximately 25% of branches showing dieback at the commencement of the survey (Figure 3); the plot located in the municipality of Alezio (GPS coordinates 40.070450, 18.043294), designated as the SS site, was the most severely affected by desiccation, with dieback exceeding 80% of the branches (Figure 4), at the moment of sampling (spring 2018). To assess potential variations in fundamental soil characteristics across the olive groves, soil samples were collected and analyzed for several key physico-chemical parameters, including soil texture, pH, cation exchange capacity (CEC), organic matter content, total nitrogen, and electrical conductivity (EC) (Table 1). Soil sampling and analysis were performed in accordance with the official methods established by the Italian Agriculture Ministry Decree *Official Methods for Chemical Analysis of Soil*, adopted since 13 September 1999 (D.M. 13 September 1999). Briefly, soil samples were collected using a soil auger at a depth of 0–20 cm. To ensure representativeness, a composite sample was created for each of the three farms by combining multiple subsamples taken in a zig-zag pattern across the orchard. The analysis followed standard procedures for determining soil pH, organic matter content, and cation exchange capacity, as outlined in the cited decree (D.M. 13 September 1999). Primary agronomic practices employed in the management of each olive grove were noted and reported (Table 2).

In May 2018, we harvested a minimum of 40 fully expanded young leaves with petioles from each sampled tree. The leaves were collected from consistent positions, with 10 leaves taken from each of the four cardinal directions. Selection criteria ensured leaves were free from mechanical damage (hail, frost, wind), parasitic attacks, and collected only from one- to two-year-old twigs exhibiting medium vegetative development, avoiding both excessively vigorous and deficient growth.

### 2.2. ICP-OES Analysis

Leaf element concentrations (Mg, Ca, K, P, Fe, Mn, Cu, Zn, and Na) were determined in oven-dried tissues (80 °C for 48 h). The dried samples underwent digestion using concentrated ultrapure nitric acid (HNO_3_, 65% *v*/*v*; Carlo Erba, Milan, Italy) in a Single Reaction Chamber (SRC) microwave digestion system. Subsequently, elemental concentrations were quantified using inductively coupled plasma optical emission spectrometry (ICP-OES) against certified multi-element standards (CPI International, https://cpiinternational.com). The limits of detection (LODs) for each element, expressed as mg kg^−1^ dry weight, were P 4.0, Mg 3.0, Cu 3.0, K 2.0, Ca 2.0, Na 1.0, Fe 0.4, Mn 0.2, and Zn 0.2.

For sulfur (S) determination, dried leaf samples (80 °C for 48 h) were ashed in a muffle furnace at 500 °C for 24 h. The resulting ash was dissolved in 10 mL of 3N hydrochloric acid (HCl) and filtered through Whatman No. 42 filter paper. The filtrate was then reacted with a 2% (*w*/*v*) barium chloride (BaCl_2_) solution to precipitate barium sulfate (BaSO_4_). Sulfur concentration was quantified turbidimetrically following the method of Bardsley and Lancaster (1960) [39].

### 2.3. Non-Protein Thiol Extraction and Determination

The quantification of non-protein thiol compounds was performed colorimetrically using 5,5′-dithio-bis-(2-nitrobenzoic acid) (DTNB), as described by Quagliata et al. (2023) [40]. In brief, frozen leaf tissues (1 g FW) were homogenized in a solution containing 80 mM trichloroacetic acid (TCA), 1 mM ethylenediaminetetraacetic acid (EDTA), 0.15% (*w*/*v*) ascorbic acid (*w*/*v*), and 1% of polyvinylpolypyrrolidone (PVPP). After centrifugation at 3500× *g* at 4 °C for 13 min, the supernatants were collected and the concentrations of DTNB-reactive compounds were detected spectrophotometrically at 415 nm (Agilent Cary 3500 UV-Vis Spectrophotometer, Santa Clara, CA, USA).

### 2.4. Enzyme Extraction and Assay and Protein Quantification

Leaf tissues (ca. 1 g FW) were powdered in a pre-chilled mortar under liquid N_2_ and then homogenized in 3 mL of a cold extraction buffer (pH 7.4), prepared with 50 mM HEPES-KOH, 5 mM MgCl_2_, 1 mM EDTA, 10% (*v*/*v*) glycerol, 0.1% (*v*/*v*) Triton X-100, 5 mM DTT, 1 mM PMSF, and 1% (*w*/*v*) PVPP. The homogenate was filtered and then centrifuged at 4 °C for 5 min at 1000× *g*. The supernatant was desalted at 4 °C on a Sephadex G-25 column (PD-10, Pharmacia, Uppsala, Sweden) pre-equilibrated with the extraction buffer without Triton X-100. The desalted extract was centrifuged at 4 °C for 5 min at 30,000× *g*, and the resulting supernatant was frozen in liquid N_2_ and stored by freezing (−80 °C) until use for in vitro enzyme assays.

O-acetylserine(thiol)lyase (OASTL; EC 4.2.99.8) activity was assayed by detecting cysteine production [41]. The reaction medium (pH 7.5) contained 50 mM Tris-HCl, 1 mM DTT, 10 mM *O*-acetyl-Ser and 2.5 mM Na_2_S. Quantitative protein determination of the leaves extracts was estimated by the protein-binding Coomassie brilliant blue G-250 dye method, using bovine serum albumin (BSA) as standard [42].

### 2.5. Statistical and Principal Component Analysis

For biochemical analyses (thiol content and OASTL activity), the data represent the average of 5 independent experiments conducted in triplicate. To identify significant differences in dependent variables induced by the growth conditions within each genotype, one-way ANOVA was followed by Tukey’s post hoc tests (*p* < 0.05) using the statistical software CoStat (version 6.45). Hierarchical clustering on ionomic datasets was performed with the full method and Euclidean distance measurement in R (V. 2023.12.0+369) using the packages pheatmap (version 1.0.12), RColorBrewer (version 1.1-3) and gplots (version 3.1.3.1). Multivariate analysis (Principal Components Analysis—PCA) was performed using the statistical software PAST (version 3.25) for Microsoft Windows 10.

## 3. Results

### 3.1. Elemental Composition of Leaves (Macro and Micro Elements)

We observed significant differences in the severity of OQDS among the olive groves, even though all trees were the same susceptible cultivar, ‘Cellina di Nardò’ (Table 3). The field in San Ligorio exhibited the lowest disease severity, with plants showing almost no symptoms (severity class 2). For clarity, we will refer to these plants as “asymptomatic” (AS). The trees in Torchiarolo showed a moderate level of symptoms (MS), corresponding to severity class 3, while the plants in Alezio displayed the most severe symptoms (SS), fitting into severity class 5.

**Figure 5:**
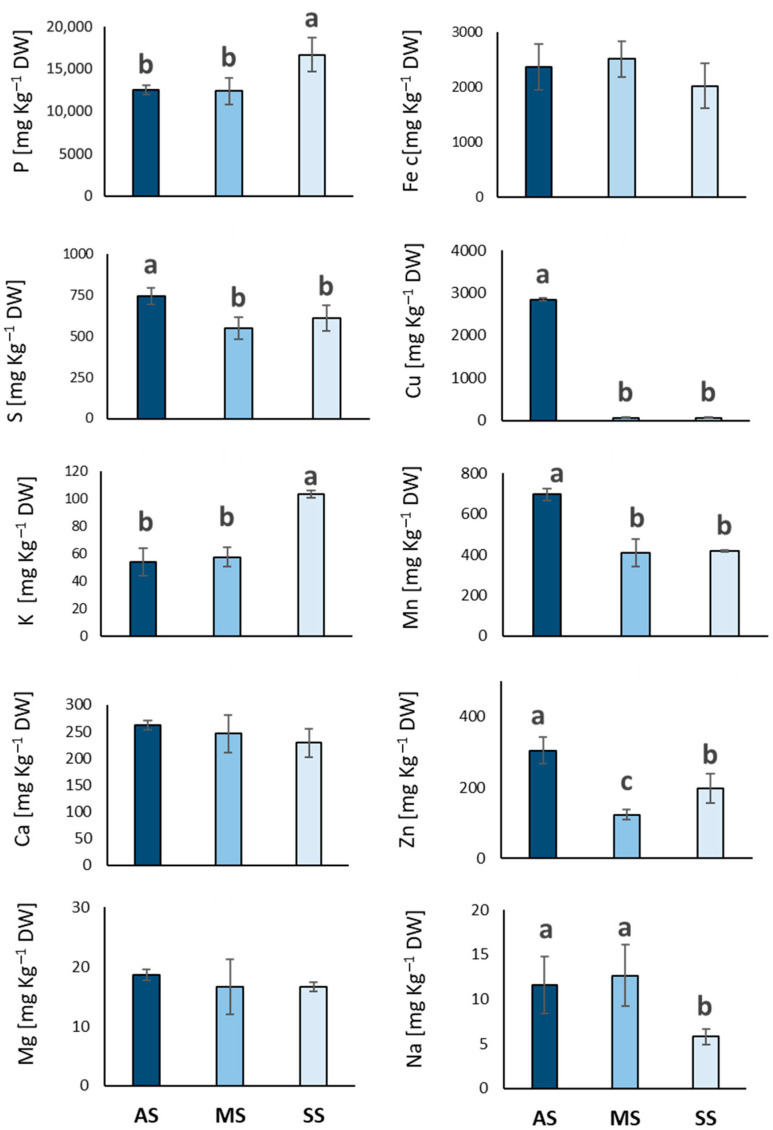
Macro- and micronutrient concentration (mg kg^−1^ dry weight) in olive leaf samples from San Ligorio (AS), Torchiarolo (MS), and Alezio (SS). The data represents the average of five independent experiments conducted in triplicate. The bars indicate the standard deviation. Different letters indicate statistically significant differences among groups (one-way ANOVA followed by Tukey’s HSD test, *p* < 0.05).

Figure 5 presents a comprehensive comparative analysis of mineral element concentrations in olive leaves collected from three experimental sites in Italy. Regarding macronutrients, phosphorus (P) levels appeared significantly higher in the SS group compared to both AS and MS (33 and 34%, respectively). Potassium (K) was also significantly higher in the SS group in contrast to the AS and MS groups (105 and 18%, respectively). Asymptomatic (AS) trees show a significantly higher S concentration compared to both MS and SS trees (35 and 21%, respectively), which showed statistically similar and lower S concentrations. Calcium (Ca) and magnesium (Mg) concentration showed no significant variation across the three groups. Examining micronutrients, manganese (Mn), copper (Cu) and zinc (Zn) were significantly more accumulated in the AS leaves compared to MS and SS. In particular, Mn concentration in AS leaves was 44 and 60% higher than MS and SS, respectively. Copper concentration was also markedly higher in the AS group compared to the very low levels observed in both MS and SS groups. Zinc (Zn) concentration was significantly higher in the AS group compared to MS and SS (145 and 53%, respectively). Sodium (Na) concentrations were significantly higher in the AS and MS groups compared to SS (99 and 118%, respectively). On the other hand, iron (Fe) and silicon (Si) levels were consistent across all three groups.

**Figure 6:**
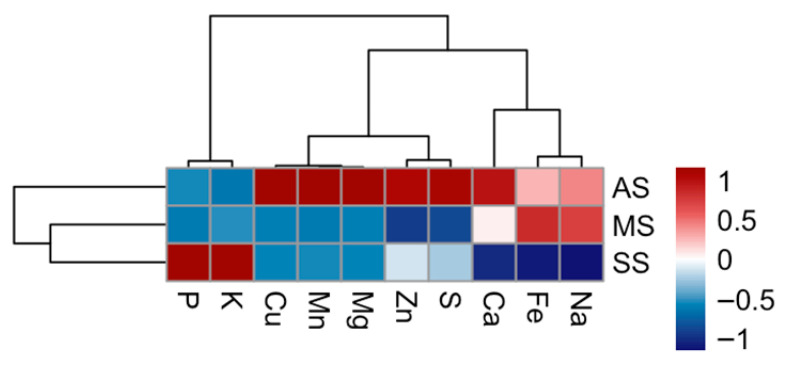
Heatmap and hierarchical clustering of macro- and micronutrient accumulation in olive leaf samples in San Ligorio (AS), Torchiarolo (MS) and Alezio (SS). Hierarchical clustering was performed to group both nutrients and samples based on similarity in their accumulation patterns, highlighting site-specific nutritional signatures potentially associated with disease severity. Color scale represents row-wise z-score values obtained after standardization of nutrient concentrations (mean = 0 for each nutrient). Red indicates values above the mean; blue indicates values below the mean.

To identify underlying patterns, hierarchical clustering has been applied to both the symptom categories and the mineral elements (Figure 6). The resulting dendrograms grouped together symptom categories that exhibit similar correlation behaviors. Notably, the asymptomatic (AS) leaves clustered alone, whereas mildly symptomatic (MS) and severely symptomatic (SS) clustered together, suggesting that they share more similar correlation patterns with the analyzed elements. Additionally, the mineral elements themselves form distinct clusters based on how their concentrations correlate with the different symptom levels. For instance, a group comprising Mg, Mn, and Cu exhibited similar trends, as did another group consisting of K and P (Figure 6).

Examining the correlation patterns across the different symptom categories reveals distinct signatures. Asymptomatic (AS) trees show a strong positive correlation with most of the analyzed elements, including Mg, Mn, Cu, Ca, S, Zn, Fe, and Na. They exhibit a negative correlation with K and P. This suggests that higher concentrations of most of the analyzed minerals tend to be associated with the absence of severe OQDS symptoms. In contrast, severely symptomatic (SS) leaves exhibit an opposite pattern. They show strong negative correlations with most of the elements that were positively correlated with AS. Interestingly, the SS category shows a strong positive correlation with K and P. This indicates that higher levels of K and P, coupled with lower levels of most other analyzed minerals, tend to be associated with severe OQDS symptoms. Mildly symptomatic (MS) leaves display correlation patterns that were generally like those of SS leaves. Similarly to the AS group, MS leaves also show a weak positive to near-zero correlation with Ca and a positive correlation with Fe and Na.

**Figure 7:**
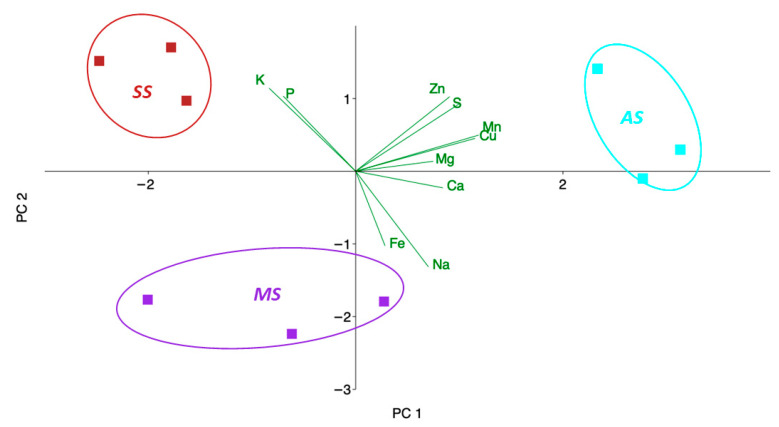
Principal Component Analysis (PCA) based on nutrient concentrations in olive leaf samples collected olive leaf samples in San Ligorio (AS), Torchiarolo (MS), and Alezio (SS). The first two principal components explained approximately 72% of the total variance. This multivariate analysis reveals distinct clustering of samples according to their nutrient profiles, reflecting site-specific nutritional patterns potentially associated with different levels of disease severity.

Figure 7 shows a Principal Component Analysis (PCA) biplot, a powerful tool for visualizing the relationships between different samples (olive trees categorized by OQDS symptom severity) and the variables (mineral element concentrations) that characterize them. The first two components used to build both the scatter and loading plots accounted for 74% of the total variance; the first component (PC1) described 49.2% of the total variance, while component 2 (PC2) described 24.45% of the variance.

Observing the distribution of the sample points, asymptomatic trees (AS) were clustered towards the positive side of PC 1. The driving variables along PC 1, those that most contribute to the separation of the asymptomatic samples from the symptomatic samples, were S, Zn, Mn, and Cu. In contrast, MS (mildly symptomatic) and SS (severely symptomatic) trees were located on the negative side of PC 1. PC 2 further differentiates the symptomatic groups. SS (severely symptomatic) trees were positioned towards the positive side of PC 2, and this separation along the second principal component appeared to be driven by the higher concentration of K and P, as indicated by their vectors pointing upwards. These higher levels of K and P in severely symptomatic trees (SS) could likely contribute to their distinction from the mildly symptomatic group (MS). MS (mildly symptomatic) trees, on the other hand, were located towards the negative side of PC 2.

### 3.2. Thiol Dynamics and S Assimilation Rate in Olive Trees with Varying OQDS Symptoms

**Figure 8:**
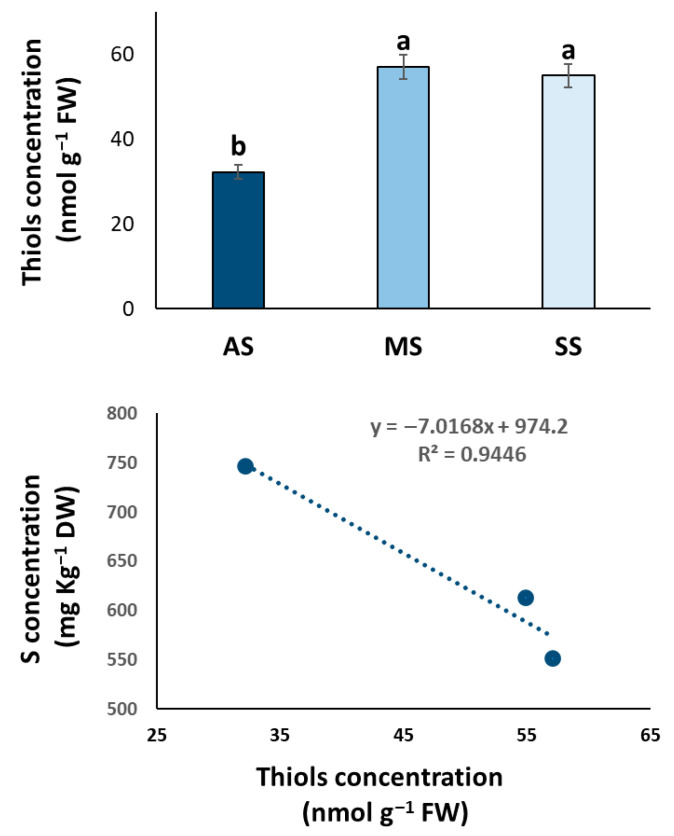
Thiol concentration and correlation with total sulfur in olive leaf samples in San Ligorio (AS), Torchiarolo (MS), and Alezio (SS). The data represents the average of five independent experiments conducted in triplicate. The bars indicate the standard deviation. Different letters indicate statistically significant differences among groups (one-way ANOVA followed by Tukey’s HSD test, *p* < 0.05).

As the PCA biplot indicated S as one of the driving variables distinguishing the asymptomatic trees, we analyzed thiol concentrations in olive leaves from the three OQDS symptom categories (AS, MS, SS) and explored the correlation between total S and thiol levels (Figure 8).

Interestingly, the pattern of thiol concentration in the three symptom categories was reversed compared to total S concentration. Asymptomatic (AS) trees exhibited a significantly lower thiol concentration compared to both MS and SS trees (56 and 58%, respectively), which showed statistically similar and higher thiol concentrations (Figure 8).

We found a clear and strong negative correlation (R^2^ = 0.9446) between total S concentration and thiol concentration, suggesting that as total S concentration increases, thiol concentration tended to decrease, and vice versa, across the different symptom categories (Figure 8).

**Figure 9:**
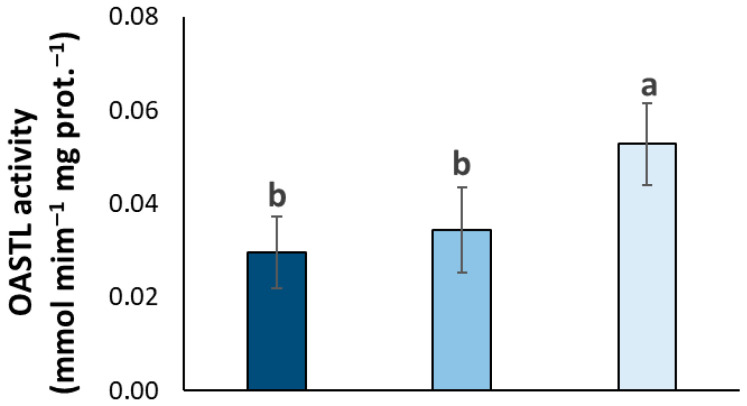
O-acetylserine(thiol)lyase (OASTL) activity in olive leaf samples in San Ligorio (AS), Torchiarolo (MS), and Alezio (SS). The data represents the average of five independent experiments conducted in triplicate. The bars indicate the standard deviation. Different letters indicate statistically significant differences among groups (one-way ANOVA followed by Tukey’s HSD test, *p* < 0.05).

To provide further insights into the observed S dynamics, we measured the activity of O-acetylserine(thiol)lyase (OASTL), the final enzyme in the S assimilation pathway responsible for the synthesis of cysteine, a precursor for various sulfur-containing compounds including glutathione (a key thiol). Data showed that OASTL activity was significantly lower in the AS and MS trees compared to the SS trees, which exhibited significantly higher activity (Figure 9).

## 4. Discussion

In the Salento peninsula, Olive Quick Decline Syndrome (OQDS) has extensively affected olive groves across three provinces. However, the spread and severity of symptoms exhibit significant heterogeneity across different areas. To unravel the complexity of this phenomenon, emerging research suggests that variations in the nutritional status and agronomical practices of olive trees may contribute to their ability to withstand infection by *Xylella fastidiosa* [7,43].

Notably, the MS and SS plots followed production techniques traditionally prevalent in the region, characterized by mineral fertilization, chemical weeding, and severe pruning. In contrast, the AS plot implemented lower-impact techniques, including more frequent but lighter pruning, the application of organic soil improvers, spontaneous grassing, and the use of foliar fertilizers and biostimulants. Some studies have already reported the role of phenolic compounds in olive leaves during *Xylella fastidiosa* infection [44]; to our knowledge, this study presents the first comprehensive investigation of olive trees of a similar age and of the same cultivar, cultivated in various fields within the OQDS-infected Salento region, yet exhibiting a spectrum of disease severity: asymptomatic (AS), mildly symptomatic (MS), and severely symptomatic (SS) states.

The analysis of the leaf ionome revealed distinct nutritional profiles associated with AS, MS, and SS states, highlighting that nutrient availability is fundamental for healthy plant growth and development and that imbalances in nutrient levels can significantly affect a plant’s ability to cope with pathogen attacks, determining the outcome of plant-pathogen interaction, resistance, or susceptibility [45].

In particular, the analysis of macronutrient concentrations revealed significantly higher levels of phosphorus (P) and potassium (K) in severely symptomatic (SS) trees compared to asymptomatic (AS) and mildly symptomatic (MS) trees. This accumulation of P and K in diseased tissues could be indicative of altered nutrient translocation or a stress-induced physiological response. For instance, the role of P content in regulating disease resistance appears to be dependent on the specific interacting plant and pathogen [46]. In *Arabidopsis thaliana*, high P supply led to P overaccumulation in leaves, accompanied by the production of reactive oxygen species (ROS), which is likely perceived by the host as a stress signal, triggering the induction of defense gene expression. Consequently, elevated P content positively regulates *Arabidopsis* immune responses, enhancing resistance to fungal pathogens [46]. Conversely, in rice (*Oryza sativa*), elevated P content has been shown to increase susceptibility to the rice blast fungus *Magnaporthe oryzae* [47]. On the other hand, P deficiency has also been implicated in enhanced resistance, as observed against *Verticillium dahliae* in cotton [48] and against insect herbivory in *Arabidopsis* [49].

These findings suggest that species-specific adaptations in P homeostasis could determine how P levels influence the complex networks governing their immune responses to pathogen attack.

The impact of K nutrition on a host plant’s susceptibility to disease can also be dualistic, conferring either an advantage or a disadvantage depending on the specific pathogen and environmental context [50]. Potassium (K) plays a pivotal role in numerous essential physiological and metabolic processes in plants, including photosynthesis, transpiration, water and nutrient transport, and disease susceptibility through its influence on enzyme regulation [51]. Enhanced K nutrition has been shown to reduce disease severity in several fungal pathosystems, including rice blast (*Magnaporthe oryzae*) in rice [52], and Septoria leaf blotch (*Septoria tritici*) in winter wheat [53]. Conversely, increased K availability has been reported to decrease disease pressure from bacterial blight (*Xanthomonas oryzae*) in rice [54].

However, the accumulation of P and K in SS trees could suggest a disruption in nutrient homeostasis caused by *Xylella fastidiosa*. As the bacterium colonizes the xylem [3,14,23], it physically blocks the translocation of essential nutrients within the plant. This blockage can lead to the observed accumulation of P and K in the leaves, indicating that these nutrients are absorbed but cannot be properly utilized or moved throughout the plant due to the compromised vascular system.

Calcium (Ca) and magnesium (Mg) are both recognized for their significant roles in plant defense [55]. Calcium, in particular, functions as a crucial second messenger, rapidly transducing signals triggered by pathogen perception and activating a cascade of downstream defense responses [56]. This signaling role suggests that Ca is a key player in the plant’s immediate response to attack. Magnesium, while not typically acting as a direct signaling molecule in defense pathways, is essential for overall plant vigor and health, impacting fundamental processes like photosynthesis and chlorophyll production. Adequate Mg levels can contribute to a plant’s ability to withstand stress and potentially influence its susceptibility to disease [57]. However, in the context of this study, the absence of significant differences in leaf Ca and Mg concentrations across asymptomatic (AS), mildly symptomatic (MS), and severely symptomatic (SS) olive trees suggests that these macronutrients may not be the primary drivers in the initial stages or the subsequent progression of OQDS symptoms within this specific olive cultivar and growing conditions in Salento. This does not exclude their importance in plant defense generally but rather indicates that other elements or metabolic shifts might be more influential in the specific dynamics of OQDS in this system. It is possible that subtle changes in Ca signaling or Mg availability at the cellular level, not reflected in total leaf concentrations, could still play a role.

Interestingly, the finding of significantly higher S levels in the leaves of asymptomatic (AS) olive trees compared to both mildly (MS) and severely symptomatic (SS) trees presents an intriguing aspect of the plant’s response to OQDS.

Sulfur is a crucial macronutrient underpinning a wide array of plant metabolic functions, including the synthesis of essential amino acids, proteins, and a diverse range of defense compounds [58,59]. Notably, research has demonstrated that appropriate S fertilization can enhance disease resistance in various plant species. Indeed, numerous sulfur-containing compounds, such as glutathione, other thiols, and glucosinolates, possess well-documented antimicrobial and antifungal properties [31]. For instance, soil application of S has shown a significant repressive effect on infections by fungal pathogens [60]: Pyrenopeziza *brassicae* in oilseed rape, *Uncinula necator* in grapes, and *Rhizoctonia solani* in potato tubers [61]. Given the established role of S in plant defense, the significantly higher accumulation of this element in the asymptomatic olive trees within our study facing the devastating OQDS warrants careful and detailed consideration.

Asymptomatic trees seem to be able to accumulate higher levels of total S, allowing overall plant health and vigor and guaranteeing future defense needs. This stored S could then be readily mobilized and incorporated into S-containing defense molecules, such as glutathione and other thiols, upon initial pathogen detection or environmental stress. However, our finding of lower total thiol concentrations in these same asymptomatic trees seems to contradict this direct mobilization theory, suggesting a more complex scenario. Sulfur exists in various forms within plants, including sulfate, cysteine, methionine, glutathione, and other more complex compounds. It is reasonable that AS trees accumulate S primarily in a storage form (like sulfate) or within metabolic pathways not directly related to immediate thiol production. This might explain the higher total S but lower thiol concentrations. When the pathogen establishes and the disease progresses in MS and SS trees, there could be a shift towards actively converting the stored S into protective thiol compounds, leading to lower total S but higher thiol levels, as found in symptomatic trees (MS and SS). Finally, the interaction with *Xylella fastidiosa* itself likely plays a significant role in influencing sulfur (S) dynamics within the olive tree. The infection and subsequent stress responses could trigger specific metabolic adjustments in the host plant, leading to alterations in S uptake, assimilation, and its partitioning within different tissues in symptomatic trees. The observed lower total S levels in these trees might be a consequence of either reduced uptake due to the pathogen’s disruption of vascular function or an increased mobilization and utilization of S in defense responses, leading to its depletion. Intriguingly, some pathogens, as highlighted by Wang et al. [59], have evolved sophisticated mechanisms to actively acquire sulfur from their host plants, potentially through the manipulation of host S metabolism pathways. Genome analysis has revealed a surprising diversity in the strategies employed by pathogens to obtain S during infection, and transcriptomic studies have uncovered variations in S uptake and metabolism patterns throughout pathogen infection cycles [59].

In contrast, micronutrient analysis highlighted a significant enrichment of Mn, Cu, and Zn in the leaves of asymptomatic (AS) trees. These micronutrients are known to play crucial roles in various physiological processes, acting as cofactors for enzymes, participating in redox reactions and electron transfer, and playing a crucial role in metabolism and stress tolerance [62,63,64]. The higher accumulation of these elements in healthy trees suggests their potential involvement in maintaining plant vigor and resilience against biotic stresses like *Xylella fastidiosa* infection. It has been reported that asymptomatic Leccino olive trees also consistently exhibited higher Mn, Ca, and Na levels in their leaves [7], consistently with our results. Notably, the markedly higher Cu concentration in AS trees aligns with previous research suggesting a potential link between Cu availability and disease development. Copper has been indeed reported to play an important, but still unclear, role in *Xylella fastidiosa* infections [65]. Similarly, the elevated levels of Mn and Zn in healthy trees could contribute to their ability to cope with the pathogen or the resulting stress. The higher Na concentrations in AS and MS compared to SS trees might reflect site-specific variations in soil salinity or uptake mechanisms, although its direct role in OQDS susceptibility remains unclear. On the other hand, the non-significant different levels of Fe across all symptom categories suggest this element might not be primary differentiating factors in this specific context.

The multivariate analyses, including hierarchical clustering and PCA, corroborated the distinct elemental signatures associated with OQDS severity. The clustering of asymptomatic trees as a separate group, distinct from the clustering of mildly and severely symptomatic trees together, underscores the unique nutritional status of healthy individuals. This suggests that the transition from an asymptomatic to a symptomatic state involves significant shifts in the leaf elemental profile. The PCA further elucidated the key mineral elements driving this separation. PC1, accounting for a substantial portion of the variance, was primarily driven by higher concentrations of S, Zn, Mn, and Cu in asymptomatic trees, effectively separating them from the symptomatic groups. This highlights the potential protective role of these elements. PC2 further differentiated the symptomatic groups, with higher K and P levels being characteristic of severely symptomatic trees. Notably, the PCA plot shows a greater separation between the asymptomatic (AS) and severely symptomatic (SS) groups along PC1, which accounts for most of the data variance. This suggests that disease progression is not a simple linear decline but a dynamic process involving different physiological stages. We suggest that the mildly symptomatic (MS) stage represents a period of active defense, where the plant metabolic response separates it from both the AS and SS groups along the PC2 axis. In contrast, the SS stage represents a state of physiological collapse where the plant defense mechanisms have failed. This terminal state, in terms of its metabolic and nutrient profile, might share a superficial similarity with the asymptomatic baseline, but it is a state of complete dysfunction. The significant separation along PC1, therefore, reflects the profound difference between a healthy plant and one that has completely succumbed to the disease, even if the intermediate stages are physiologically complex.

Prompted by the PCA identifying S as a key differentiating element, our investigation into S metabolism revealed a complex picture. Asymptomatic trees exhibited significantly higher total S concentrations, but paradoxically lower total thiol levels compared to symptomatic trees. This inverse relationship was further supported by a strong negative correlation between total S and total thiol concentrations across the symptom categories. To understand this dynamic, we analyzed the activity of OASTL, a crucial enzyme in cysteine biosynthesis, the precursor of thiols. The significantly lower OASTL activity in asymptomatic and mildly symptomatic trees compared to the higher activity in severely symptomatic trees provides a potential explanation for the observed thiol levels. In healthy trees, a lower demand for immediate stress response might lead to lower OASTL activity and thus lower thiol accumulation, despite a higher overall S content potentially stored in other forms. Conversely, the upregulation of OASTL in severely symptomatic trees likely reflects an increased need for protective thiol compounds like glutathione to combat the stress induced by *X. fastidiosa* infection, potentially leading to a depletion of total S pools for this purpose.

In conclusion, our findings demonstrate that OQDS is associated with significant alterations in the leaf elemental composition of olive trees. Asymptomatic trees exhibit a nutritional profile characterized by higher concentrations of specific micronutrients (Mn, Cu, Zn) and total S, coupled with lower thiol levels and OASTL activity. Conversely, severely symptomatic trees accumulate higher levels of phosphorus and potassium while showing lower concentrations of several micronutrients and higher thiol levels and OASTL activity. These results suggest that maintaining adequate levels of specific micronutrients could be crucial for olive tree health and resilience against OQDS. Furthermore, the altered S metabolism, characterized by a trade-off between total S and thiol accumulation depending on the disease stage, warrants further investigation to fully understand the defense mechanisms and metabolic shifts occurring in response to *X. fastidiosa* infection.

Future research should focus on elucidating the specific roles of these key elements and sulfur compounds in the OQDS pathosystem and exploring potential nutritional management strategies to mitigate disease severity [19].

## Figures and Tables

**Table 1:** Soil analysis.

		AS	MS	SS
Sand (2.0–0.05 mm)	%	66	73	69
Silt (0.05–0.002 mm)	%	16	14	14
Clay (<0.002)	%	18	13	17
pH		7.8	7.5	7.6
Cation Exchange Capacity (CEC)	meq/100 g	13.70	10.19	13.45
Organic Matter	%	2.18	1.41	1.34
Total Nitrogen	%	0.13	0.090	0.077
Electrical Conductivity (EC)	mS/cm	0.303	0.363	0.330

**Table 2:** Notable agronomic practices and olive orchard management for the infected fields in San Ligorio (AS), Torchiarolo (MS), and Alezio (SS) (2012–2018).

Practice	AS	MS	SS
Pruning	Every two years	Every four years	Every two years
Soil fertilization	N-P (25-15) (every two years); organic amendments (every two years)	Urea (every two years)	NPK (15-5-5) + Fe-Zn (every two years)
Foliar fertilization	Zn-Mn-Cu; protein hydrolysate	Mg-B-Cu-Fe-Mn-Zn	-
Chemical weeding (Glyphosate)	-	Every two years	Every year
Chemical weeding (Oxyfluorfen)	-	Every year	-
Antifungal treatments	-	Copper Oxychloride	Copper Sulfate
Pesticide treatments	-	Pyraclostrobin; Dimethoate	Dimethoate

**Table 3:** Disease severity and class of sampled trees. This table details the disease severity of sampled olive trees, with symptoms evaluated in September 2017. Trees were classified using a scale from 1 to 5, as follows: 1 = no wilted branches; 2 = slightly wilted branches (up to 25% of the canopy); 3 = generally wilted branches (from 26 to 50% of the canopy); 4 = severely wilted branches (from 51 to 75% of the canopy); 5 = highly severe wilted branches (over 76% of the canopy).

Sampled Tree	Wilted Branches	Severity Class	Average Severity (Corresponding Class)
AS1	4%	2	4% (2)
AS2	0%	1	
AS3	13%	2	
AS4	1%	2	
AS5	2%	2	
MS1	58%	4	27% (3)
MS2	36%	3	
MS3	28%	3	
MS4	11%	2	
MS5	2%	2	
SS1	75%	4	81% (5)
SS2	84%	5	
SS3	87%	5	
SS4	76%	5	
SS5	83%	5	

## Data Availability

The original contributions presented in this study are included in the article.
